# Application of Wireless Accelerometer Mounted on Wheel Rim for Parked Car Monitoring

**DOI:** 10.3390/s20216088

**Published:** 2020-10-26

**Authors:** Michal Borecki, Arkadiusz Rychlik, Arkadiusz Olejnik, Przemysław Prus, Jan Szmidt, Michael L. Korwin-Pawlowski

**Affiliations:** 1 Institute of Microelectronics and Optoelectronics, Warsaw University of Technology, 00-662 Warszawa, Poland; j.szmidt@imio.pw.edu.pl; 2 Faculty of Technical Sciences, University of Warmia and Mazury in Olsztyn, 10-719 Olsztyn, Poland; rychter@uwm.edu.pl (A.R.); arkadiusz.olejnik@uwm.edu.pl (A.O.); 3 Blue Oak Inventions, 56-400 Wroclaw, Poland; prus.przemyslaw@gmail.com; 4 Département d’informatique et d’ingénierie, Université du Québec en Outaouais, Gatineau, QC J8X 3X7, Canada; michael.korwin-pawlowski@uqo.ca

**Keywords:** car wheels monitoring, safety of car use, attack on car wheels, 3D accelerometer application, car rim wireless sensing, adaptive signal classifier

## Abstract

Damages of different kinds that can be inflicted to a parked car. Among them, loosening of the car wheel bolts is difficult to detect during normal use of the car and is at the same time very dangerous to the health and life of the driver. Moreover, in patents and publications, only little information is presented about electronic sensors available for activation from inside of the car to inform the driver about the mentioned dangerous situation. Thus, the main aim of this work is the proposition and examination of a sensing device using of a wireless accelerometer head to detect loosening of wheel fixing bolts before ride has been started. The proposed sensing device consists of a wireless accelerometer head, an assembly interface and a receiver unit. The assembly interface between the head and the inner part of the rim enables the correct operation of the system. The data processing algorithm developed for the receiver unit enables the proper detection of the unscrewing of bolts. Moreover, the tested algorithm is resistant to the interference signals generated in the accelerometer head by cars and men passing in close distance.

## 1. Introduction

Owning a car is associated with ensuring the correct preparation for driving, driving the car and parking it. A preparation for driving is related to the condition of the car and driver. The preparation of the car includes checking the technical condition of the car and the supply of adequate technical fluids, including brake fluids [1] and fuel of the correct quality [2]. The preparation of the car driver, in the most simplified terms, consists of the approval of its functional state [3,4]. The mentioned preparations could be supported by the use of independent sensors and systems. Correct technical conditions while the car is driven are assured by an onboard diagnostic system called OBD [5].

Currently, the safety of car driving can be supported by the use of a variety of sensing systems. The classic sensing systems increasing the safety of car operation include: tire-pressure monitoring system (TPMS), adaptive cruise control (ACC), blind-spot detection (BSD), lane-departure warning (LDW), traction control (TC) sometimes called rollover prevention, antilock braking system (ABS), emergency brake assist (EBA), adaptive headlights and rearview camera. All these systems are well developed, but the progress in technology and system performances are under continuous improvements [6,7,8,9,10,11].

### 1.1. Motivation of Project Undertaking

It is important to realize that not all vehicles damage comes from crashes that occur at high-speed collisions as a result of driver lack of concentration or precision. Some driving car accidents may result as an effect of external factors, such as blinding the driver, car brakes failure, or the wheel falling off. The blinding of the driver is common at night due to oncoming traffic with high beam on or with badly set lights. The car brakes failure or the wheel falling off may be a result of malicious damages made to a parked car.

It should be noted that parked cars are vulnerable to all types of natural, vandalism and malicious damages, as well as to unauthorized use or theft [12]. A significant contributor to parked car attack is the way the car is parked [13]. Typical car vandalism acts include car being scratched, smashed wing mirrors, windows or lights, snapped windscreen wipers, graffiti, slashed tires or damaged rims. Malicious car vandalism includes braking lines damage and loosening of the wheels.

In certain neighborhoods the problem of tampering with the wheels of parked cars is a real one. So is on certain parking lots where long-distance truckers park overnight. The concerns of those communities, perhaps not very well appreciated in affluent suburbs where cars are kept overnight in garages, were at the origin of the project. Moreover, presented problem of car wheel bolts unscrewing is real one in Eastern Europe, but its genesis is not clear to everyone.

It should be noted that it is usually by monitoring the distance driven that the brake monitoring system (BMS) monitors the linings of the friction surfaces for wear, while real-time brake efficiency monitoring systems are based on accelerometers [14]. At present, brake mechanical reliability [15,16] and its functions as braking support in different situations are in the mainstream of development [17,18,19]. It has been established that pressure sensors mounted in brake-fluid reservoir or brake fluid presence sensors can allow the detection of the early stage of brake system failures [20]. Moreover, the functional state of braking pipes or lines can be monitored with fiber-optic transmission line implemented as optical continuity sensor or wire break sensors [21,22]. In future, the braking by wire technology that is under development may solve the problem of consistent monitoring of brake lines [23].

Unauthorized by the owner changes of the car’s position may be a result of a parked car being pushed, or of theft [24,25]. Common car theft is realized with breaking into the car and car’s ignition system. The stolen car leaves, driven by its own engine [26]. Car pushing is realized with the engine off. The position of a car can be detected with such systems as the Global Positioning System (GPS), Global Navigation Satellite Systems (GNSS), that uses wireless technology of communication via the Global System for Mobile Communications (GSM) including the Narrowband Internet of Things (NBIoT) [27,28,29]. But, positioning of objects in high urban areas is not a trivial task [30,31]. Therefore, additional information of initial car movement when being passed to the city monitoring system, may be useful [32].

### 1.2. Modern Methods of Parked Car Protection

Since no one can predict or prevent those types of property damages or theft, the insurance policy to cover the costs is often considered [33]. Comprehensive insurance coverage, or parked car insurance can be useful in some situations. However, after a vandalism act, the car and comprehensive coverage policy owners have to fill a claim that usually results in a partial refund of repair costs limited by a deductible and also results in future increased premiums for the insurance policy. Moreover, an insurer often requires guilt indication even though the perpetrator who made intentional damage is usually not known [34]. Thus, car vandalism prevention is connected with dash camera installed in a parked car security system. These implementation critical issues are ensuring a continuous power supply and ensuring sufficient memory for recording of events. The parking mode of dash camera operation may include event trigger recording, time-lapse recording and buffered recording coupled with event trigger. An event triggering the recording could be an impact detected by the accelerometer mounted inside the dash camera, or a quick change in the monitored image that is detected by the software implemented in the dash camera. It is important to realize that impact and motion detection can be deceived by random wind blast as well as by the motion of objects in a busy area. Therefore, the security systems for parked car provide the integration of different electronic units, as for example, tilt sensor with open circuit sensor of doors and hood or accelerometer with dash camera. The integration is realized by dedicated data processing which eliminates false alarms.

On the other hand, malicious car damages can be often detected with simple methods. The braking system damage can be seen by visual inspection of the ground under the parked car. The loosening of wheels bolts can be detected with a torque wrench. However, routine use of these methods may be impractical and look ridiculous in some situations. It should be noted also, that dash-camera area of observation does not include the wheels area.

### 1.3. Accelerometers and Their Application in Car Monitoring

Accelerometers are well developed sensors that can be used to detect the characteristic movement of the body of a car. Accelerometers have been used for monitoring vehicle dynamics and driving characteristics [35,36]. Because the sensing heads are often installed in the sprung part of the vehicle, obtained results of acceleration are influenced by the spring and damping forces. The interesting case of accelerometer use for monitoring the driving characteristics is accelerometers mounted on the wheel hubs [37].

In some computer science works, such accelerometers are described as of the smartphone type [38]. But the most common accelerometers used in mobile application are classified as micro-electro-mechanical systems (MEMS) [39]. Therefore, progress in application of accelerometers in cars monitoring is correlated with microelectronics technology development [40] as well as with data processing and sensors integration for working in hostile environments [41]. It should be noted that some commercial companies offer small-dimension units that integrate accelerometers supported with wireless transmission to a remote unit, as for example the iNode Care Sensor family. The producer offers on its web site three accelerometer circuits LIS3DH, MMA8452 or MMA8451 [42] and the iNode Care Sensor electronic board integrated with a housing and battery for push-fit assembly. In real world, precise placing and mounting of accelerometers on the monitored object is not a trivial task [43]. For car wheel monitoring, mounting of accelerometers usually was considered on the shaft frame [44] as well as in different positions on wheel rims [45]. Among the issues to consider for accelerometer car monitoring is the road condition and traffic characteristics [46]. Moreover, the accelerometer head can be used to detect vibrations which are represented by subtle acoustic signals that are distributed in car rim [47].

### 1.4. The Aim of the Work

Based on the analysis of the state of the art, it can be concluded that the loosening of the car wheel bolts is difficult to detect during normal use of the car and is at the same time dangerous to the health and life of the driver. Moreover, in patents and publications, only little information is presented about electronic sensors that are available to implementation inside of the car and inform the driver about the mentioned dangerous situation. Thus, the main aim of this work is the proposition and examination of a sensing device that consist of a wireless accelerometer head, positioned on rim, and a receiver with implemented algorithm to detect the loosening of wheel fixing bolts before the ride has been started. The real challenge is to develop an algorithm that: -ensures proper classification of signals, -is insensitive to disturbing signals, -results in acceptable energy consumption in the head-mounted on the rim. The sensor head positioning on the rim also introduces challenges. The mass of the sensor head has to be limited to an acceptable value. Challenges assumptions for the development of wireless sensor head construction are; -the mass of the head has to be lower than the standard TPMS sensor, -the time of head continuous working has to be longer than seven days, -the montage and disassembly process of the head on the rim is simple.

## 2. Construction of a Sensing Device with the Head of Wireless Accelerometer Mounted on Wheel Rim

The typical construction of the sensing device described as the wireless accelerometer includes two units: the head of wireless accelerometer and the receiver unit.

### 2.1. Head of Wireless Accelerometer

**Figure 1:**
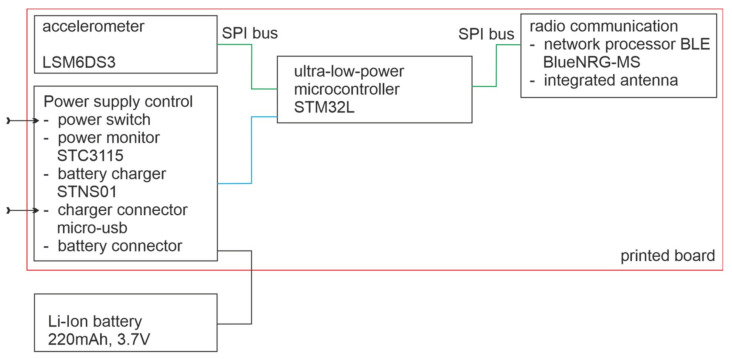
Schematic diagram of head of wireless accelerometer (WA).

In this paper, the head of wireless accelerometer (WA) was made with the LSM6DS [48] chip that was connected with microcontroller STM32. The microcontroller was also connected to peripheral circuits of power supply and data transmission. The schematic diagram of the head of wireless accelerometer is presented in Figure 1.

The LSM6DS chip can be configured for operating in few measurement modes: the low-power, normal mode and high-performance mode. These modes differ mainly by internal sampling frequency of accelerometer transducers. For further examinations the normal mode with 104 Hz of internal sampling frequency has been selected. Measurement results are collected in the internal buffer before being transmitted to the microcontroller and the wireless connection. Data transmission to the receiver unit is programed with Blue Tooth Low Energy technology (BT). Due to the transmission characteristics, it should be expected that some data will be lost the in real environment. It should be noted that battery powering of remote head is the second drawback of wireless applications. Rechargeable battery powering adds the consideration the parameter of the expected time between charging. For practical purpose the time of 7 days of permanent use has been established as the objective. Those requirements can be satisfied with a 220 mAh capacity battery when the repetition rate of data acquisition and transmission is below 100 Hz.

**Figure 2:**
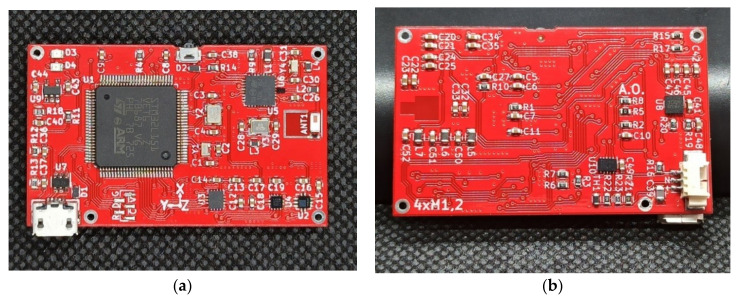
The printed board of the head of wireless accelerometer: (**a**) Upper side; (**b**) Bottom side.

The view of the printed board of the wireless accelerometer is presented in Figure 2.

At the upper side of the printed board, the microcontroller STM32, the USB connector, the power switch and the antenna are easily visible (Figure 2a). At the bottom side is the battery connector. The printed board is attached to the case with four M1.2 × 2.5 mm screws. The case was made using the 3D printing technology from Nebula PET-G material. The case was also used to integrate the Li-Pol 220 mAh, 3.7 V battery, the dimensions of which are 36 × 20 × 4 mm. It should be noted that the battery electrical performance can be affected by road-induced vibrations [49]. Therefore, the battery was mounted with the double-side self-adhesive acrylic foam tape from 3M. The covers of the power switch and the micro-USB connector were printed with the use of TPU Flex 40D from Print-me. The total weight of the head of the wireless accelerometer is 30 g which is comparable with a standard tire pressure sensor. For example, the weight of the Mobiletron universal TX-C001 TPSM module is 46 g.

**Figure 3:**
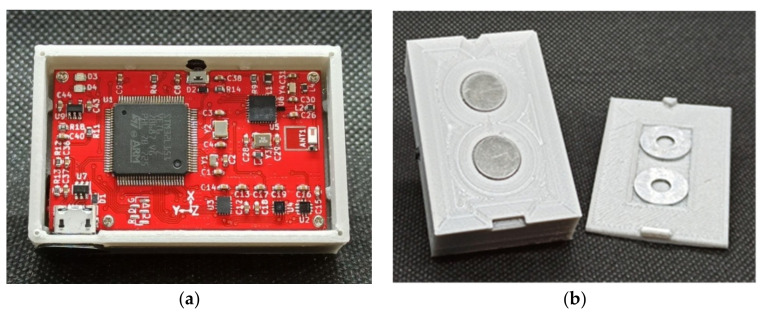
The case of head of wireless accelerometer: (**a**) The printed board assembly in its case; (**b**) The magnetic link: -bottom side of case at left, -upper side of sled at right.

The issue of head positioning on the rim was solved using a magnetic link. Two small magnets were secured to the bottom of the head’s case with chemical-resistant adhesive transfer tape 96105CR [50]. The dedicated sled interface between the head and the rim was designed. The sled body was printed. The sled shape was almost flat at the upper side and curved at the bottom. At the upper side of the sled a recess and directional tabs have been designed. Directional tabs ensured an unambiguous placement of the accelerometer head. The recess was used to mount two steel disks. The assembly was done with the same tape as used for magnets positioning. The radius of curvature of the lower part of the sledge coincides with the inner radius of curvature of the wheel rim. The sledge assembly was done with the use of double side adhesive transfer tape type 950 from 3M. In this way a reliable and separable connection of the head with the rim was achieved. The case of wireless accelerometer with printed board position as well as the sled interface are presented in Figure 3.

**Figure 4:**
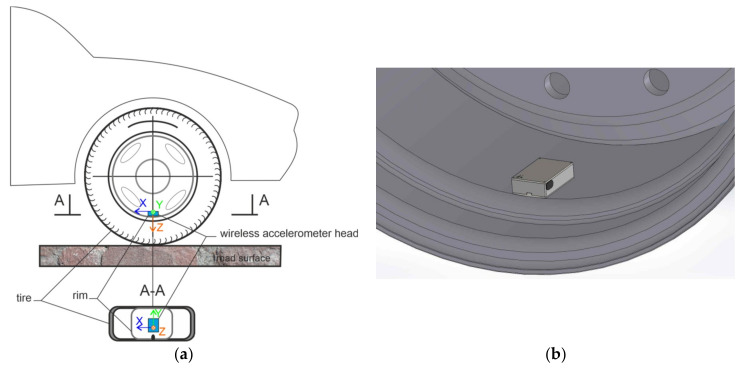
Positioning of the head of wireless accelerometer on rim: (**a**) Scheme of monitored acceleration directions; (**b**) Scheme of the head mounted on the outer side of the wheel rim with the use of the sled.

**Figure 5:**
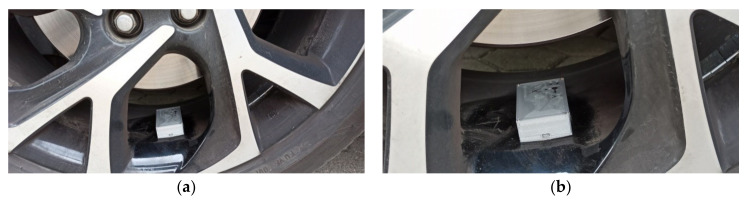
The head of wireless accelerometer (WA) view that is used in examinations: (**a**) Distant view; (**b**) Close view.

In this work the accelerometer was mounted on rim according to the scheme presented in Figure 4, while the view of the placement is presented in Figure 5. The distant view shows the dimensional comparison between rim and the wireless accelerometer head, while the close view shows the monitored acceleration axes and position of the power switch.

### 2.2. Receiver for the Wireless Accelerometer

In this paper, the receiver for the wireless accelerometer was made with the use of the tablet running on the Android system. The software enables a data to text file recording that includes time stamp of measurements and three axis acceleration values. Most of the data were received, but some data loss occurred. If the data is not received the mark “nan” instead of a number value is inserted in the file. Data receiving periodicity is set to 97 Hz. In all measurements the tablet was positioned inside the car and the wireless head was mounted on the rim. Standard acceleration of free fall, sometimes abbreviated as standard gravity, used by the accelerometer as unit of measurement results is denoted in this paper as g_0_.

## 3. Wireless Accelerometer Verification with Laboratory Equipment

**Figure 6:**
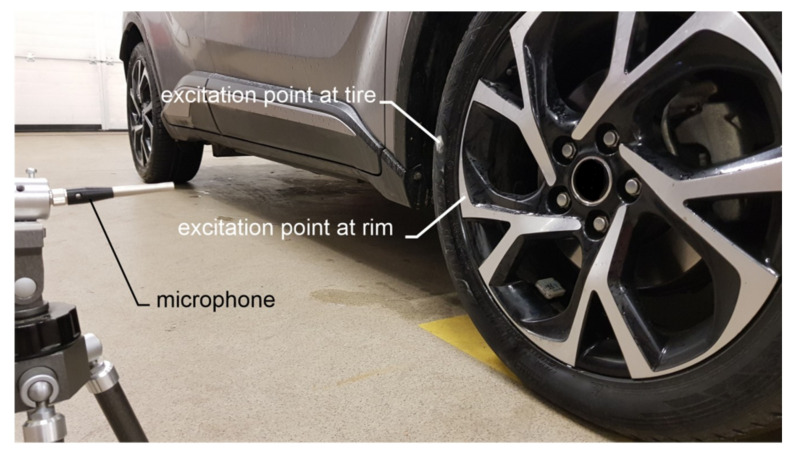
Measurement set-up for vibration analysis -points of excitations of vibrations.

Laboratory verifications of the wireless accelerometer were made as comparative measurements of accelerometers answer to rim and tire stroked by the hammer and wheel wrench. The roofing hammer weighing 250 g as well as wheel wrench of a cross-type were used to induce vibrations. Verifications by the acoustic method were made with ROGA RG-50 microphone connected to KSD-400 vibration analyzer. The sampling frequency of acoustic pressure was set to 2048 Hz. The microphone was set in line with the axis of car wheel at 0.5 m distance. The wireless accelerometer was set to vibration measurement mode. Two points of excitations of vibrations were selected; one positioned on the rim, the second positioned on the tire, as presented in Figure 6.

**Figure 7:**
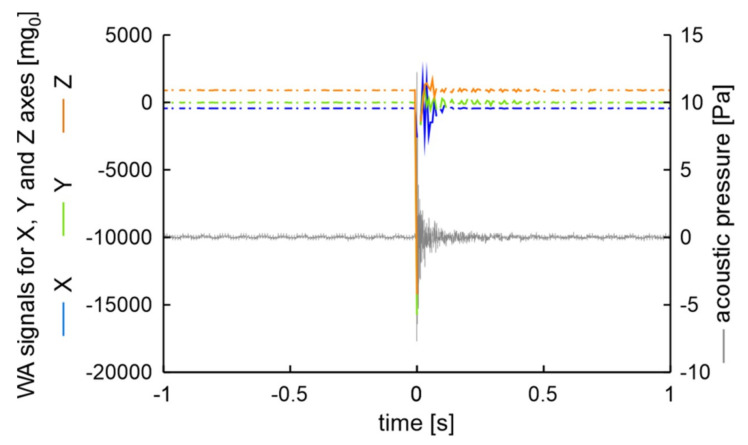
Examination results when the excitation was made on rim.

Examination results when the excitation was made on the rim are presented in Figure 7. For measurement synchronization, the moment of excitation was assumed as 0 s.

**Figure 8:**
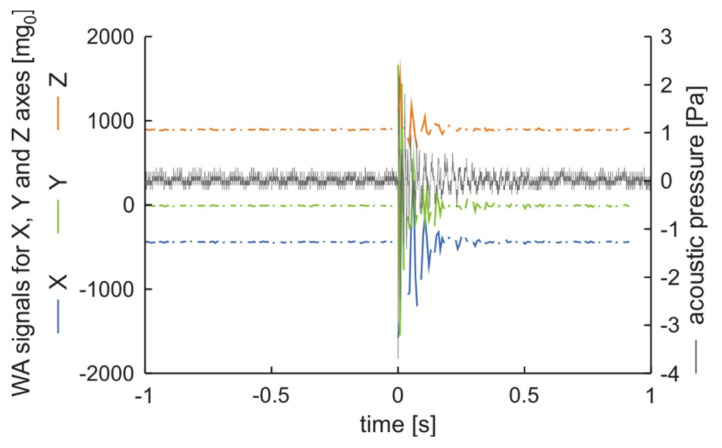
Examination results when the excitation was made on the tire.

The measurement results prove that the applied excitation shows in acoustic vibration analysis and in all accelerometer channels. After vibration excitation, in both methods, the signals rise faster than they decrease. The time series of signals are similar in both methods. It should be noted that due to lower sampling frequency of the accelerometer than of the vibration analysis system, the signals of accelerations are more asymmetrical in relation to ambient signal level. Examination results when the excitation was made on the tire are presented in Figure 8. Received signal values now are lower in all channels that in the previous measurement. Vibrations damping now is also lower than in aforementioned measurement. The analysis of Figure 7 and Figure 8 leads to the conclusion that the measurement results from the wireless accelerometer are similar to the results of acoustic examination.

## 4. Analysis of Wireless Accelerometer Working Conditions

The wireless accelerometer is intended to work at a car parked and its working results are indicative of an attack on the car wheel. Therefore, two types of signals are present in measurements. The first type is of signals occurring in normal situations. The second type is of signals occurring during an attack on the wheel by unscrewing the bolts. In all experiments the car which wheels were monitored was parked with the hand brake engaged. In this chapter, for simple visual data analysis, the wireless accelerometer head in all examination was in position as or very close to that presented in Figure 4a.

### 4.1. Accelerator Signals Occurring in Normal Situations

**Figure 9:**
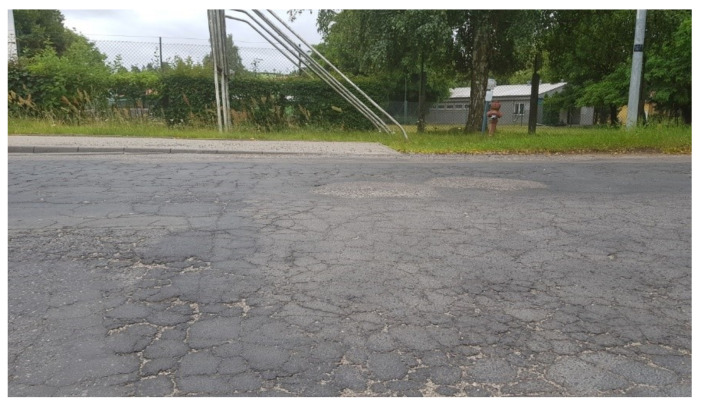
Worn road surface (WRS) used in research, photographed from the point of view of a parked car.

**Figure 10:**
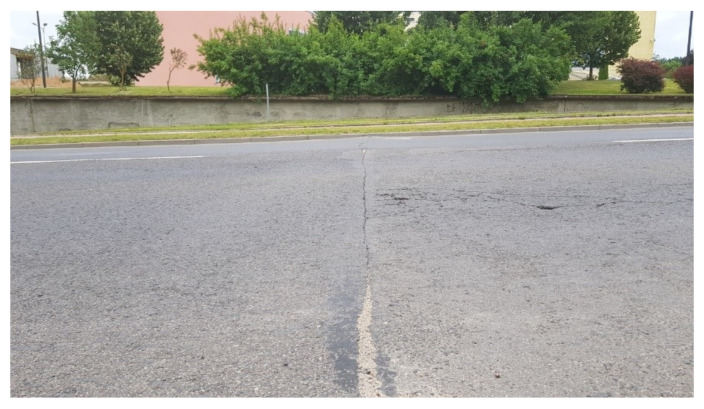
Single crack perpendicular to the direction of road (SCP), photographed from the point of view of a parked car.

Signals occurring in normal situations may be the result of an approaching person, but typically they are results of another car, van, bus or truck moving at close distance to the parked car. In standard situations, the transmission of vibration excitation is realized by air as well as by a common surface. Excitation of vibration by air is popularly called air blow. The generated intensity of air blow can be related to the object’s speed and dimensions. The road surface can act at some range as a source and transmission medium of surface acoustic waves. The generated intensity of surface acoustic waves may be linked to excitation and propagation parameters. Therefore, the object’s weight and the type of road surface should be also taken under considerations. There are two typical cases when the road is the source of acoustic waves to other objects. The first is a worn surface as presented in Figure 9. The second is a single crack perpendicular to the direction of a road as presented in Figure 10. To reduce the number of analyzed cases, the presented roads were in an area with the same speed limit of 50 km/h. During these examinations vehicles moved at different speeds. Vehicles on the worn road surface moved not faster than 40 km/h. On the flat road surface with a single crack perpendicular to the lane road the speed limits were often exceeded. One vehicle’s speed was about 60 km/h. Only the speed of a city bus was slower than the limit and was 40 km/h.

**Figure 11:**
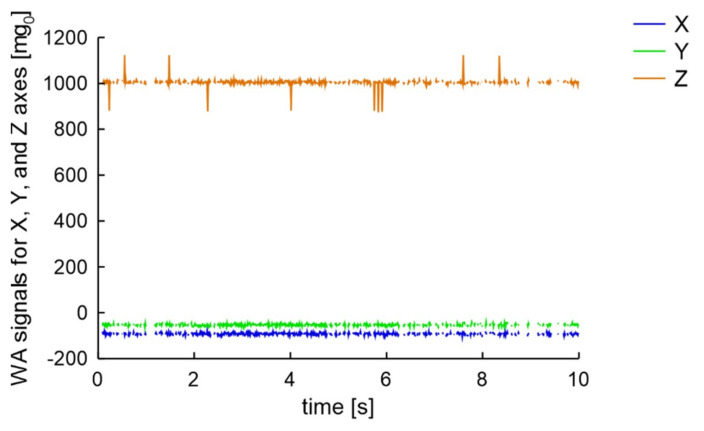
Measurement results for a man walking on WRS in 1 m distance from parked car.

The first examination was performed for a man that was walking on the worn road surface at 1 m distance from the parked car. The results are presented in Figure 11. The trend values of accelerations are near 0 mg_0_ at the X and Y axis as well as 1000 mg_0_ at the Z-axis. The noises are similar on all axes and do not excess ±10 mg_0_. The signal on Z-axis sometimes presents peaks value with a local amplitude of 120 mg_0_. These peaks appeared when a man steps on the worn road surface. The results show that the acoustic wave generated by a man walking is detected by the sensor only in the Z-axis is in agreement with the expected properties of acoustic waves.

**Figure 12:**
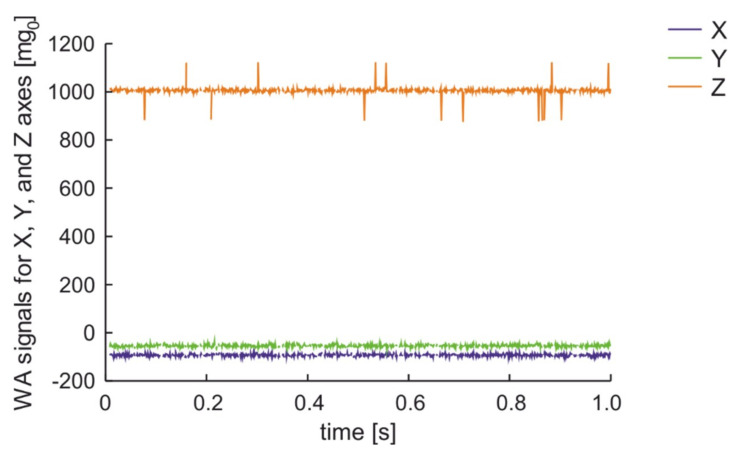
Measurement results for a van and city bus crossing with 40 km/h over on WRS in 2 m distance from parked car.

The second experiment was a comparison of signals generated by a van with a mass of about 2000 kg on the two presented road surfaces. As the experiment include real data, for the worn road surface case the van was followed by a city bus. The distance between the monitored parked car and the passing vehicles was about 2 m. The results, presented in Figure 12, confirm that both vehicles on worn road surface generate in the wireless accelerometer a signal only in the Z-axis.

**Figure 13:**
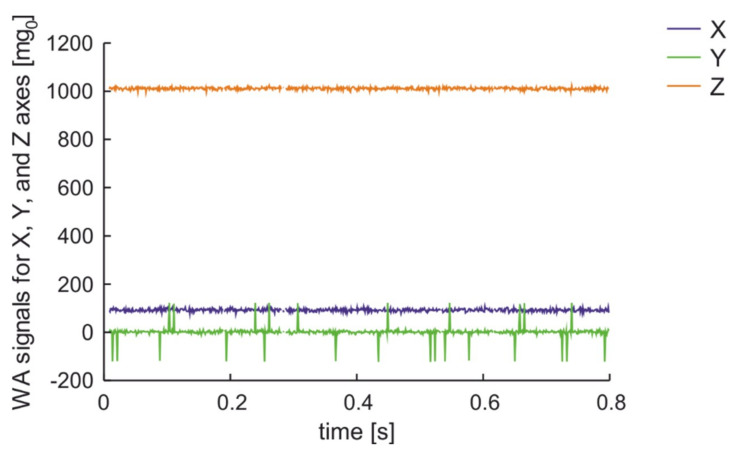
Measurement results for a van moving on SCP with 60 km/h in 2 m distance from parked car.

A similar van going at 60 km/h over the almost flat surface of the road with single crack perpendicular to the direction of road excites the signal in the wireless accelerometer presented in Figure 13. Similar results were obtained for passenger cars. In those cases, the excitation of vibration occurs only in Y-axis that is in the plane of the road and is perpendicular to the vehicle’s moving direction, as shown on Figure 4a.

**Figure 14:**
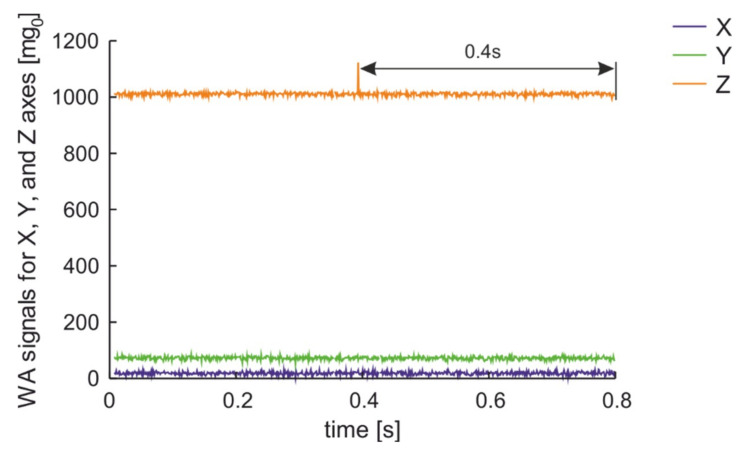
Measurement results for a city bus moving on SCP with 40 km/h in 2 m distance from parked car.

A city bus that goes at 40 km/h over the same road as the previous van generated the signal presented in Figure 14. The bus had two axles, but signals from a single axle were visually detected as 0.4 s of observation results in 4.4 m distance of movement which is less than distance between bus axles. In this case the excitation of vibrations occurs only in Z-axis.

**Figure 15:**
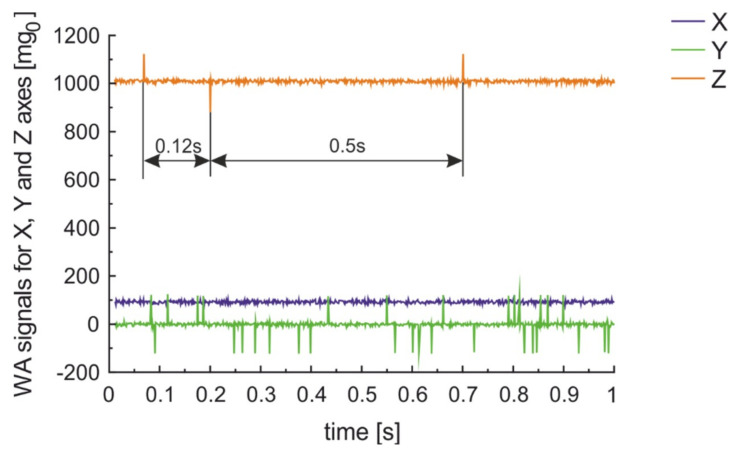
Measurement results for a truck moving on SCP with 40 km/h in 1 m distance from parked car.

Truck similar in size to city bus, but characterized by three axles with wheels equipped with standard quality tires crossing on SCP road with speed of about 40 km/h generated results presented in Figure 15. The signal from all three axles are visible. The distance between axles calculated on measured time and speed looks rational 11 m/s × 0.5 s gives 5.5 m between front and rear axle. The moving truck seems very noisy. Further examination shows that city bus was equipped with tires that are ranked with 70 dB noise while truck tires are ranked as 74 dB of noise.

As could be assumed, the speed of the moving vehicle is the dominant factor in generation of air blow that results in Y-axis vibration excitation of the parked car. Moreover, the vehicle mass and surface roughness are responsible for acoustic wave generation that results in Z-axis excited vibrations. The distance from the parked car to a source of acoustic surface wave excitation also is important. All excited vibrations result in the local increase of measured acceleration lower than 130 mg_0_. It should be noted furthermore that in all situations the X axis excitations are less than the electric noise level.

### 4.2. Acceleration Signals Occurring at Unscrewing the Wheel Bolts

**Figure 16:**
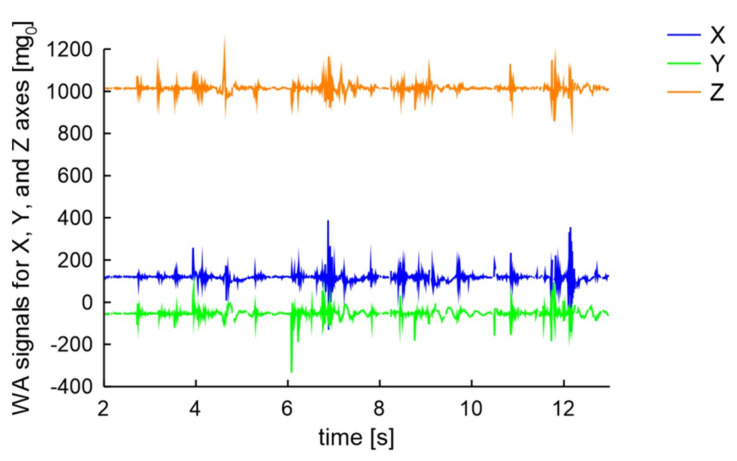
Signal of 4 wheel-bolts being unscrewed using the X type key.

Acceleration signals occurring generated by unscrewing wheel bolts depend on many factors. Typical signals of 4 bolts being unscrewed using a cross type key are presented in Figure 16.

Unscrewing the bolts generates signal in all axes. Its amplitude is a little higher and the local peaks are denser compared to those generated by moving vehicles in close distance. But, the direct conclusion of bolts being unscrewed based on raw signals is somehow confusing.

## 5. An Algorithm for Indication of Unscrewing of the Wheel Bolts

**Figure 17:**
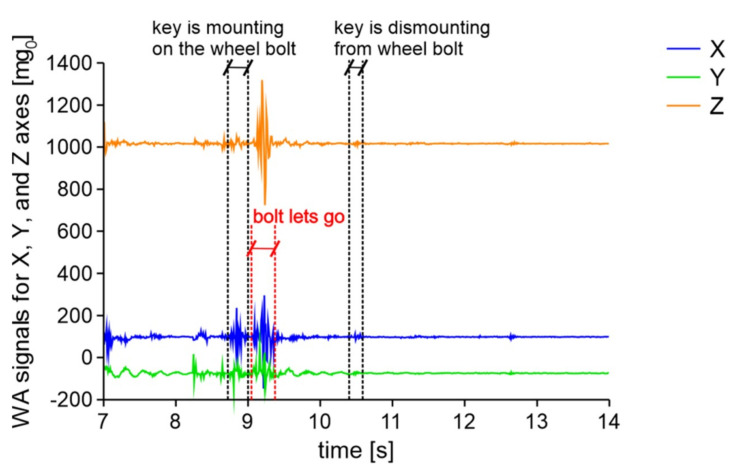
Signal of a man unscrewing a single wheel bolt and goes away in laboratory conditions.

The unscrewing process of the wheel bolt consists of three steps. First, the key is mounted on the wheel bolt. Next, the wheel bolt is unscrerved. Last, the key is unmounted. The whole process made by a trained man can last below 1 s. The situation in laboratory conditions when a man is unscrewing a single wheel bolt and goes away is presented in Figure 17. The signal observed in Figure 17 shows that unscrewing a wheel bolt is observed by the sensor as a collection of peaks. These peaks maximum amplitudes are in the range from 200 to 300 mg_0_, thus the values are comparable but higher than disturbing signals. Fortunately, the density of peaks is much greater in case of the bolt unscrewing than the typical disturbing signals that come from normal situations as can be seen in Figure 11, Figure 12, Figure 13, Figure 14 and Figure 15.

In the ideal situation of sensor head positioning presented in Section 4.1, when the axes are in classic position, the signals in X-axis are not sensitive to disturbing signals. In real situations, when the sensor head is in an arbitrary position, signals collected in all axes are affected by disturbing signals and signals that come from bolt unscrewing. Signals at X and Y axes are sensitive to key montage. But, at beginning of the bolt unscrewing wheel has sometimes a little tendency to rotate, thus additional signal in X ax is generated. When the bolt lets go the signals are generated on all three axes. But, the examination of a signal of the single axis may in the future reduce the power consumption of the accelerometer head. Therefore, signal from X has been selected for further detection algorithm development.

### 5.1. Algorithm Proposition

It is important that in case of unscrewing a wheel bolt of a parked car signal changes are generated in the X-axis. These signal changes are separated from distributing signals of objects that are passing in close distance to monitored car only in the case when sensor head is in upper and lower position of wheel. Thus, detection of unscrewing wheel bolt requires dedicated signal processing and using of signal qualifier.

The first step of proposed algorithm is simply ignoring missing data as the volume of these data is lower than 5% in the measurement period of 1 s. This can be written as:

IF (*x*_i_ == *nan*) THEN: (reject *x*_i_) and (*i*--), (1)

where: *x*_i_ actual signal at time indexed by *i*, *nan*—not a number.

As the position of the head is not constant, the measured signal is under influence of the component of gravitation acceleration. Fortunately, for parked cars the component of gravitation acceleration is constant, therefore it can be removed from analyzed signals as the initial measured value when the wireless accelerometer is activated. Therefore, the initial step of signal processing is removing of the constant component according to:

*n*_i_ = *x*_i−_*x*_0_, (2)

where: *x*_0_ is signal at sensor activation, *n*_i_-signal at moment i with removed initial component.

**Figure 18:**
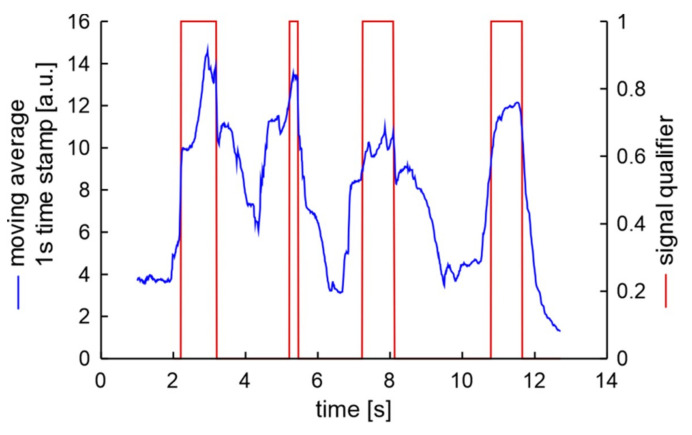
Processed signal of unscrewing 4 bolts.

Next step of signal processing is converting the signal values to absolute values. This conversion enables signal processing with the moving average method. The method enables to differentiate signals with different density peaks. To enable proper signals differentiation the moving average time stamp is here 1 s and is related to typical unscrewing time of wheel bolt:

*ma*_j=0_ = 0, 1 s = 97 (samples), FOR (*i* = *j*-1 s; TO *i* = *j*) DO: *ma*_j_ = *ma*_j_ + |*n*_i_|, ma_j_ = ma_j_/1 s, (3)

where: *ma*_j_—moving average of normalized module of signal at moment *j*, *j* > 1 s. Such processed signal of unscrewing 4 bolts is presented in Figure 18. The measurement data of first second is not presented as in this time the moving average increases from 0 due to its initial wake-up time. Next second of measurement from 1.0 s to 2.0 s is used to calculate and store of noise level signal. This signal value is almost stable at 4 units.

The result of processed data series can be described as four peaks. These peaks are not smooth, not of equal height and not of equal width, but they are well developed and are related to unscrewing of each bolt. It can be seen that unscrewing of the second bolt started faster than next two bolts. The common characteristic these peaks is the increase of the signal of at least 5 units in 1 s. Therefore, signal processing can be written as:

*h* = 0, *sq*_j_ = 0, IF (*ma*_j_ < 5 + *ma*_j+1s_) THEN: (*sq*_j+1s_ = 1), ELSE: (*sq*_j+1s_ = 0), IF (*sq*_j_ == 1 and *sq*_j+1s_ == 0) THEN: *h* = 1, ELSE: *h* = 0, (4)

where: *sq*—signal qualifier at defined moment, *h*—variable that indicate potential hazard of sq. Such form of increase serves as qualifier better than a static qualifier for accurate detection of wheel bolts unscrewing when during the unscrewing operation the wheel is a little rotated. Therefore, such qualifier of processed signal of bolt unscrewing has been selected. The deactivation of signal qualifier of bolt unscrewing requires additional time for damping hazard signals. In the presented study 0.1 s was selected:

IF (*h* == 1) THEN: [FOR (*j* = *now* TO *j* = *now* + 0.1 s) DO: *sq*_j_ = 0]. (5)

The equations from (1) to (5) describe proposed algorithm. The algorithm has been used for analysis of data presented in Section 4.1. A false screw loosening signal was not detected.

### 5.2. Algorithm Verification

The proposed algorithm was verified with experiments that cover different possible situations. Two different mechanics were unscrewing the bolts on two different cars with two different mechanical keys, a cross type and pipe wrench type. Car number S1 was regularly serviced. Car number S2 was serviced randomly. The full experiment plan is presented in Table 1. Both cars have 5 bolts on the wheel. Each of the wheels of S1 car were equipped with a single lock nut. Wheels of the S2 car were mounted with standard bolts. Therefore, for S1 car the test for unscrewing 4 bolts was performed and the test for unscrewing 5 bolts was performed for car S2. Moreover, the test for S1 car was realized on a street parking, while the test for S2 car was performed in the laboratory. Thus, eight different examinations were performed.

The full list of examinations, according to the notation proposed in Table 1, can be written as {S1AX, S1AL, S1WX, S1WL, S2AX, S2AL, S2WX, S2WL}.

**Figure 19:**
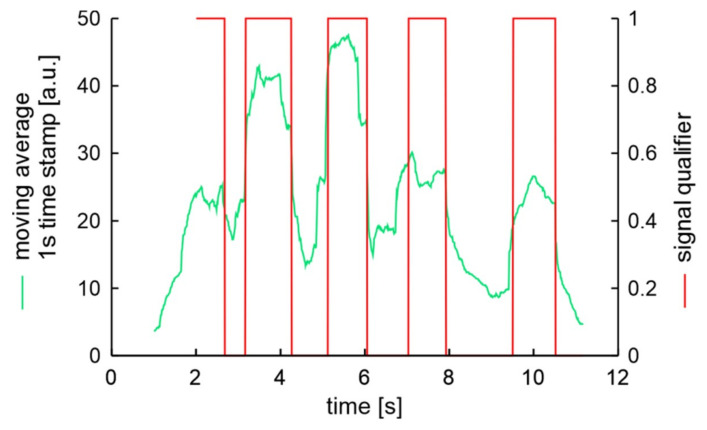
Processed signal of unscrewing 5 bolts in experiment S2AX.

The first and reference examination that was presented in Figure 18, can be described as S1AX. The verification experiment result described as S2AX, which means the same mechanic was unscrew bolts in a different car with the use of the same key, is presented in Figure 19.

In this case, unscrewing was started just after the sensor was activated and was as fast as possible. In the presented situation the moving average characteristic values are higher than in the previous case. The peaks are very close one to another in such a way that minimal values of valleys are significantly greater than the expected signals of accelerometer noises. But it should be noted that noise levels visible at the beginning and at the end of the experiment are similar to those in the case presented in Figure 18. Despite the speed of mechanic procedure all five bolts unscrewing was detected.

**Figure 20:**
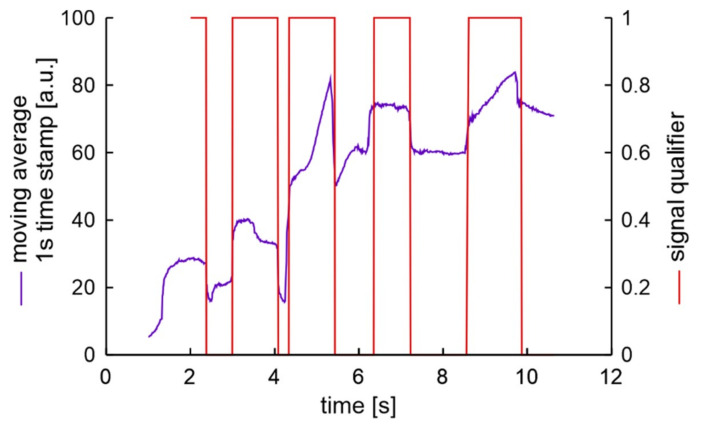
Processed signal of unscrewing 5 bolts in experiment S2WL.

Data (S2WL) obtained during wheel unscrewing by a mechanic W for a car with inactive handbrake and slightly rusty bolts are presented in Figure 20. The moving average tendency of increasing value is visible and is the expected result of wheel movement. The qualifier works flawlessly detecting the unscrewing of all 5 bolts.

**Figure 21:**
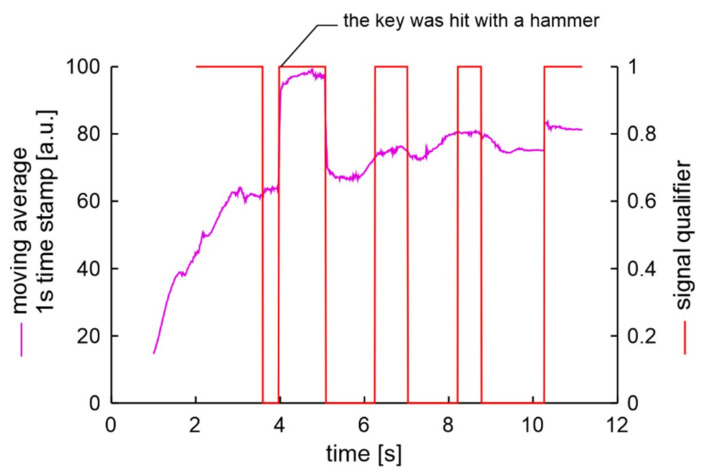
Processed signal of 5 bolts unscrewing in experiment S2AL.

The most rusted bolts were unscrewed in the S2AL experiment. During unscrewing of the second bolt the key was stopped by bolt. In order to release the key, it was hit with a hammer, as presented in Figure 21. Still, the algorithm shows correctly the bolts unscrewing process.

All other experiments {S1AL, S1WX, S1WL, S2WX} gave correct and similar results.

## 6. Conclusions

The proposed wireless accelerometer sensing device with assembly interface and receiver unit shows its ability to classify signal of unscrewing of car wheel bolts. The assembly interface and wireless head construction are crucial for proper signal measurement. Moreover, the electronic circuit construction including assembly interface provides the possibility of car wheel monitoring in real environment with the presence of electromagnetic noise. The proposed data processing algorithm reduces classification errors to an acceptable minimum as proven in a verification experiment performed for 8 different situations in which there was no false alarms. Initial digital signal normalization and data processing with moving averaging of function parameters referred to standard time of bolts unscrewing enables accurate automatic classification. Thus, the real challenge of developing an algorithm that: ensures proper classification of signals, is insensitive to disturbing signals, and results in acceptable energy consumption in the head-mounted on the rim has been achieved. Moreover, this work shows that single axis acceleration monitoring is enough to classify bolts loosening. This open a way for next generation device development, that can work a much longer on single battery charge.

Challenges assumptions for the development of wireless sensor head construction are achieved also: the mass of the head is 30 g with housing and battery and is lower than the standard TPMS sensor that mass is 46 g, the time of head continuous working is longer than seven days, and the montage and disassembly process of the head on the rim is realized with the magnetic link.

The aim of planned future works is an extension of the test situations set and carrying out extensive tests and construction modification for the improving of wireless head time of work so that the head may work 6 months on a single battery charge.

## Figures and Tables

**Table 1:** Experiment plan for algorithm verification.

Car Number	Mechanic First Letter of Name	Mechanical Key Type	Place of Examination
S1	A	X	parking place beside street
S2	W	L	laboratory
